# Coping with depression and anxiety in Egyptian physicians during COVID-19 pandemic

**DOI:** 10.1186/s43045-020-00070-9

**Published:** 2020-11-11

**Authors:** Ola Osama Khalaf, Mohamed A. Khalil, Reham Abdelmaksoud

**Affiliations:** Psychiatry Department, Faculty of Medicine, Cairo University, Cairo, Egypt

**Keywords:** COVID-19, Healthcare, Physicians, Depression, Anxiety, Stress, Coping, Egypt

## Abstract

**Background:**

The COVID-19 pandemic is a public health emergency with a negative impact on mental health. Healthcare workers are one of the most vulnerable groups to psychological stress in pandemics especially COVID-19. In this cross-sectional study, we assessed depression, stress, and coping among a sample of Egyptian physicians using an electronic survey. It included demographic data; Depression, Anxiety and Stress Scale-21 Items (DASS-21); and Brief Resilient Coping Scale (BRCS).

**Results:**

We found that the majority of the sample were females (61.2%), in medical specialties (51.2%), and living with vulnerable family members (92.4 %). The majority (63%) suffered from severe or extremely severe depression, 77.6% had extremely severe anxiety, and 72% suffered from stress. BRCS showed that only 17.1% had high resilient coping. Female physicians had significantly higher depression, anxiety, and stress scores of DASS than male physicians (*p* = 0.001, < 0.001, and < 0.001, respectively). The anxiety scale was significantly higher in those with chronic diseases (*p* = 0.040) while the stress scale was lower significantly in those with higher academic degree (*p* = 0.034). Age had a significantly negative correlation with DASS anxiety (*p* = 0.031) and stress scores (*p* = 0.037). The BRCS score had a significantly negative correlation with the depression, anxiety, and stress scales of DASS (*p* = 0.018, 0.014, and 0.007 respectively).

**Conclusion:**

The COVID-19 pandemic has a negative impact on the psychological well-being of the studied Egyptian physicians. Prophylactic measures should be implemented to avoid development of psychiatric symptoms in physicians.

## Background

In January 2020, the WHO classified the coronavirus disease 2019 (COVID-19) pandemic as a public health emergency [13].

Emergencies in public health including pandemics are known to have a negative impact on mental health at different levels [15]. At the individual level, it causes fear, helplessness, and stigma. As for communities, psychiatric morbidity may increase similar to what happened in SARS outbreak in 2003 [16].

Such emergencies threaten health and safety creating a state of insecurity and unpredictability. In the SARS outbreak, healthcare workers suffered from fears of being infected, infecting family/friends, stigma, and high levels of stress, anxiety, and depressive symptoms [11]. This is evident in the COVID-19 pandemic due to many factors: limited knowledge and resources, unavailable treatment, conflicting media messages, and social distancing. Healthcare workers are one of the most vulnerable groups to psychological stress in pandemics. Moreover, with COVID-19, healthcare workers suffer from longer working hours and scarce personal protective equipment (PPE) [15]. In addition, they are challenged with deficient resource allocation to equally critical patients and rather impossible balance between their own needs being understaffed with the expanding number of patients. These pressures are intensified by time urgency and public and media scrutiny [6, 19].

Many studies assessed factors mediating psychiatric morbidity during pandemics. This includes profession (doctor/nurse), marital status, presence of social support, training competency, and coping mechanisms [7].

Coping is an important mediator between stress and mental illness such as anxiety and depression [4].

The literature on COVID-19’s effect on mental health is currently expanding but is still limited. In this study, we aim at assessing the depression, anxiety, and stress among a sample of physicians in different specialties in Egypt and also determining their ability to cope with these stresses. We assume that COVID-19 has a negative impact on the psychological health of physicians. Additionally, we assume that proper coping skills neutralize the effect of COVID-19.

## Methods

This was a cross-sectional study using a convenient sample. An anonymous survey was distributed among doctors through social media via link. The link was sent to doctors’ groups of specific specialty or sent individually. The survey was time-limited to 3 months and was carried out from March to May 2020 during the COVID-19 pandemic.

The survey was written in English and was titled Survey among Medical staff. It started with a must-answer question about whether or not the candidate would like to participate or not.

The questionnaire included demographic data; the Depression, Anxiety and Stress Scale-21 Items (DASS-21) [12]; and Brief Resilient Coping Scale (BRCS) [17].

We included physicians from both genders and different ages and years of experience. Physicians included were working in governmental general hospitals. They treated patients with COVID-19 from different medical complications or comorbidities according to their specialty. History of psychiatric disorders or treatment with psychotropics was excluded by history taking.

Clinical specialties were clustered into 3 categories: surgical, medical, and supportive. Supportive group of specialty includes microbiology, nuclear medicine, pathology, radiology, anesthesiology, clinical genetics, and radiotherapy according to the Dutch classification [3].

Demographic data included age, gender, marital status, academic level, occupation, specialty, and working years.

Some questions were added to assess the risk to COVID-19 to self (like suffering from chronic illness) and to others (living with vulnerable groups). A question to assess workload (working hours/week during last month) was added.

The DASS-21 consists of 3 self-report scales that assess depression, anxiety, and stress during the past 7 days. The depression scale assesses dysphoria, hopelessness, devaluation of life, self-deprecation, lack of interest, anhedonia, and inertia. The anxiety scale assesses autonomic arousal, skeletal muscle effects, situational anxiety, and subjective experience of anxious affect. The stress scale assesses difficulty relaxing, nervous arousal, and being easily upset/agitated, irritable/overreactive, and impatient. Responder has to choose between 4 answers ranging from “did not apply at all” to “apply very much.” Higher scores indicate severity. Scores for the three scales are calculated by summing the scores for the relevant items, and the severity of each scale is defined (normal, mild, moderate, severe, or extremely severe) (Table 1) [12].

**Table 1:** DASS interpretation and categories

DASS interpretation	Depression	Anxiety	Stress
Normal	0–9	0–7	0–14
Mild	10–13	8–9	15–18
Moderate	14–20	10–14	19–25
Severe	21–27	15–19	26–33
Extremely severe	28+	20+	34+

Coping was assessed using the Brief Resilient Coping Scale. It is a standardized 4-item scale that evaluates the resilience and coping to stressors. Responders have 5 choices in each question: does not describe me at all, does not describe me, neutral, describe me, or describe me very well. Higher scores indicate higher resilience. The total score classifies responders to low, medium, and high resilience copers (Table 2) [10].

**Table 2:** BRCS interpretation and categories

BRCS interpretation	Score range
Low resilient copers	4–13 points
Medium resilient copers	14–16 points
High resilient copers	17–20 points

Data were analyzed using the Statistical Package for the Social Sciences (SPSS) version 20 [9]. Mean and standard deviation were used for describing the numerical data while count and frequency described the categorical data. Comparisons between 2 groups were done by Student’s *t* test and chi-square test. One-way ANOVA was used for comparing 3 groups. Association between numerical groups was done by Pearson’s correlation test.

There was no missing data.

## Results

One hundred and seventy physicians participated in the survey over the 3-month period. Two thirds of them were females (61.2%) with mean age of 36.5 years. Other demographic data are shown in Table 3.

**Table 3:** Demographics and clinical characteristics of the physicians

Physicians (***N*** 170)		***Number/frequency***
**Age in years** *(mean ± SD)*		36.47 ± 5.08
**Gender**	Males	66/38.8%
	Females	104/61.2%
**Marital status**	Single	43/25.3%
	Married	123/72.4%
	Divorced or widow	4/2.4%
**Academic degree**	Bachelor	9/5.3%
	Master	66/38.8%
	MD	95/55.9%
**Job**	Resident	17/10.0%
	Assistant lecturer	38/22.4%
	Lecturer	57/33.5%
	Associate professor	16/9.4%
	Professor	10/5.9%
	Other	32/18.8%
**Specialty**	Surgical specialties	44/25.88%
	Medical specialties	87/51.17%
	Supportive specialties	39/22.94%
**Years of experience**	Less than 5 years	14/8.2%
	5–10 years	49/28.8%
	More than 10 years	107/62.9%
**Working hours per week** *(mean ± SD)*		27.36 ± 25.67
**Living with vulnerable family members**	No	13/7.6%
	Yes	157/92.4%
**DASS**^a^ **depression score** *(mean ± SD)*		12.54 ± 6.72
**DASS depression**	Normal	12/7.1%
	Mild	18/10.6%
	Moderate	33/19.4%
	Severe	44/25.9%
	Extremely severe	63/37.1%
**DASS anxiety score** *(mean ± SD)*		14.44 ± 7.37
**DASS anxiety**	Normal	9/5.3%
	Mild	7/4.1%
	Moderate	10/5.9%
	Severe	12/7.1%
	Extremely severe	132/77.6%
**DASS stress score** *(mean ± SD)*		11.58 ± 6.98
**DASS stress**	Normal	49/28.8%
	Mild	20/11.8%
	Moderate	35/20.6%
	Severe	33/19.4%
	Extremely severe	33/19.4%
**BRCS**^b^ **score** *(mean ± SD)*		13.45 ± 2.95
**BRCS interpretation**	Low resilient copers	85/50.0%
	Medium resilient copers	56/32.9%
	High resilient copers	29/17.1%

^a^*DASS* Depression, Anxiety and Stress Scales

^b^*BRCS* Brief Resilient Coping Scale

Physicians in medical specialties were 51.2% of total participants; meanwhile, surgeons were 25.88% and supportive specialty physicians were 22.94%. Physicians worked 27.36 h per week in average. The majority of them (92.4%) were living with vulnerable family members (Table 3).

The depression scale of DASS was 12.54 ± 6.72 in which 63% of physicians had either severe or extremely severe depression and only 7% of them were normal on this scale. Meanwhile, the anxiety scale was 14.44 ± 7.37 and 77.6% of physician had extremely severe anxiety. Twenty eight percent of physicians were normal on stress score (Table 3).

The Brief Resilient Coping Scale score was 13.45 ± 2.95. Half of physicians were low resilient copers, one third of them were medium resilient copers, and 17.1% were high resilient copers (Table 3).

Female physicians were significantly higher in the depression, anxiety, and stress scales of DASS than male physicians (*p* = 0.001, < 0.001, and < 0.001, respectively). The anxiety scale was significantly higher in those with chronic diseases (*p* = 0.040) while the stress scale was lower significantly in those with higher academic degree (*p* = 0.034). Marital status, specialty, years of experience, and living with a vulnerable family member did not show significant differences in DASS scores (Table 4).

**Table 4:** Relation between demographics and clinical characteristics of the physicians

		***N***	DASS^a^ depression		DASS anxiety		DASS stress		BRCS^b^ score	
			Mean ± SD	***p***	Mean ± SD	***p***	Mean ± SD	***p***	Mean ± SD	***p***
**Gender**	Males	66	10.42 ± 5.98	0.001	11.92 ± 6.54	< 0.001	9.01 ± 6.53	< 0.001	13.59 ± 3.10	0.615
	Females	104	13.88 ± 6.84		16.04 ± 7.45		13.21 ± 6.79		13.35 ± 2.87	
**Marital status**	Single	43	11.37 ± 5.83	0.421	14.16 ± 6.98	0.927	11.16 ± 6.19	0.883	13.42 ± 2.78	0.805
	Married	123	12.93 ± 7.05		14.50 ± 7.59		11.75 ± 7.34		13.49 ± 3.02	
	Divorced or widow	4	13.00 ± 4.24		15.50 ± 5.92		11.00 ± 2.83		12.50 ± 3.32	
**Academic degree**	Bachelor	9	17.44 ± 6.61	0.079	19.33 ± 8.45	0.123	17.44 ± 7.25	0.034	12.55 ± 2.87	0.542
	Masters	66	12.22 ± 5.29		14.22 ± 5.50		11.36 ± 5.83		13.66 ± 2.65	
	MD	95	12.29 ± 7.45		14.12 ± 8.26		11.18 ± 7.49		13.38 ± 3.16	
**Specialty**	Surgical specialties	44	12.31 ± 6.28	0.838	14.14 ± 6.99	0.885	11.59 ± 6.88	0.967	13.20 ± 2.65	0.405
	Medical specialties	87	12.40 ± 6.75		14.38 ± 7.57		11.47 ± 7.16		13.32 ± 3.23	
	Supportive specialties	39	13.10 ± 7.23		14.92 ± 7.50		11.82 ± 6.84		14.00 ± 2.60	
**Years of experience**	Less than 5 years	14	14.50 ± 6.51	0.523	16.79 ± 8.10	0.382	14.14 ± 7.22	0.275	12.57 ± 2.44	0.515
	5–10 years	49	12.45 ± 6.66		14.78 ± 6.86		11.96 ± 7.06		13.53 ± 3.11	
	More than 10 years	107	12.33 ± 6.79		13.98 ± 7.50		11.07 ± 6.89		13.52 ± 2.95	
**Living with vulnerable family members**	No	13	12.08 ± 5.59	0.796	15.23 ± 8.80	0.689	11.00 ± 5.94	0.755	12.85 ± 1.91	0.447
	Yes	157	12.58 ± 6.82		14.38 ± 7.27		11.63 ± 7.07		13.50 ± 3.02	
**Chronic disease**	No	135	12.07 ± 6.08	0.150	13.85 ± 6.94	0.040	11.11 ± 6.31	0.163	13.52 ± 2.87	0.537
	Yes	35	14.34 ± 8.62		16.71 ± 8.57		13.40 ± 8.99		13.17 ± 3.30	

^a^*DASS* Depression, Anxiety and Stress Scales

^b^*BRCS* Brief Resilient Coping Scale

The Brief Resilient Coping Scale score did not show significant differences in different categories of gender, marital status, academic degree, specialty, years of experience, living with vulnerable family members, and chronic diseases (Table 4).

Age had a significantly negative correlation with DASS anxiety (*p* = 0.031) and stress scores (*p* = 0.037). Weekly working hours were not significantly correlated with any of DASS scores (Table 5).

**Table 5:** Correlation between Depression, Anxiety and Stress Scales; Brief Resilient Coping Scale; and age

		DASS^a^ depression	DASS anxiety	DASS stress	BRCS^b^ score
**Age**	*r*	− .147	− .166	− .160	.075
	*p*	.056	.031	.037	.334
**Weekly working hours**	*r*	.008	− .082	− .027	.063
	*p*	.916	.300	.729	.425
**DASS depression**	*r*		.890	.923	− .182
	*p*		< 0.001	< 0.001	.018
**DASS anxiety**	*r*	.890		.916	− .188
	*p*	< 0.001		< 0.001	.014
**DASS stress**	*r*	.923	.916		− .206
	*p*	< 0.001	< 0.001		.007
**BRCS score**	*r*	− .182	− .188	− .206	
	*p*	.018	.014	.007	

^a^*DASS* Depression, Anxiety and Stress Scales

^b^*BRCS* Brief Resilient Coping Scale

The Brief Resilient Coping Scale score had a significantly negative correlation with the depression, anxiety, and stress scales of DASS (*p* = 0.018, 0.014, and 0.007, respectively) (Table 5).

## Discussion

Our study describes the psychological impact and mental health of the medical staff in a convenient sample of Egyptian physicians.

The majority of physicians had either severe or extremely severe depression while 77.6% of them had extremely severe anxiety. Anxiety was significantly higher in those with chronic diseases; this has been proven by several researches that depression and anxiety occur with chronic diseases [2] but also the underlying chronic disease such as hypertension, respiratory system disease, and cardiovascular disease may be risk factors in severe COVID-19 patients compared with non-severe ones [20]; this may rise the anxiety among medical staff members who suffer from chronic illness.

Unexpectedly, the weekly working hours were not significantly correlated with DASS. This can be explained by the precautionary measures against COVID-19 which included decreasing the workforces in some specialties (apart from frontline healthcare physicians) resulting in a wide range of working hours for our sample. In addition, the unusual situation related to COVID-19 outbreak may be the main reason for higher score scores of DASS regardless of the workload.

Stress was found to be less among higher education level. This might be explained and understood that senior physicians are less exposed as they have fewer working hours than junior one and more experienced in dealing with critical situations. Moreover, seniors are elder and age was found to be inversely correlated to anxiety and stress scales of DASS. As age advances, the personality becomes stable and less confused under stress as persons become comparatively free of neurotic anxiety [14].

Further analysis for the results showed significant difference between males and females as regards levels of depression, anxiety, and stress as measured by DASS-21 and also as regards resilience as measured by BRCS. This higher symptom prevalence in females resonates well with results from surveys conducted in other countries [18, 20] and is also similar to a Chinese study conducted on 246 medical staff during the COVID-19 pandemic, the incidence of anxiety in female medical staff was higher than that in male, and the score of self-rating anxiety scale in female medical staff was higher than that in male [8]. Women usually show more reactivity than men in neural networks associated with fear and arousal responses [5].

As regards the significantly positive correlation between the triad of depression, anxiety, and stress, it could be explained that all of them have similar pathophysiology where there are abnormalities in the regulation of the hypothalamic-pituitary adrenal axis and the sympatho-adrenomedullary system [1].

The significant negative correlation between this triad and resilience coping was suggested by previous researchers who found that coping may play an important role in mediating the outcomes of stressful events, including anxiety, depression, and other psychological distress [4].

## Conclusion

Therefore, we can conclude that the psychological well-being of the studied Egyptian physicians in this sample is affected negatively by the COVID-19 pandemic suffering from depressive, anxiety, and stress symptoms. These results should raise our attention to the medical staff and their mental health status, so we recommend more prevention efforts such as screening for mental health problems, psycho-education for stress management strategies and acquiring healthy coping skills (setting a daily routine, avoiding too much news about COVID-19), and psychosocial support.

Limitations of this study include the relatively small sample size, and the whole data were self-rated which may limit the data generalizability. Also, the effect of direct contact with COVID-19 patients was not studied. We recommend in future studies increasing the staff sample size and categorizing the experiences based on profession.

## Data Availability

All data analyzed during this study are included in this published article.

## Abbreviations

ANOVA: Analysis of variance
BRCS: Brief Resilient Coping Scale
DASS-21: Depression, Anxiety and Stress Scale-21 Items
PPE: Personal protective equipment
SARS: Severe acute respiratory syndrome
SPSS: Statistical Package for the Social Sciences
WHO: World Health Organization
